# Effect of sliding friction in harmonic oscillators

**DOI:** 10.1038/s41598-017-03999-w

**Published:** 2017-06-16

**Authors:** Miguel V Vitorino, Arthur Vieira, Mario S Rodrigues

**Affiliations:** 1 Biosystems & Integrative Sciences Institute, Faculdade de Ciências, Universidade de Lisboa, 1749-016 Lisboa, Portugal; 2 Departamento de Física, Faculdade de Ciências, Universidade de Lisboa, 1749-016 Lisboa, Portugal

## Abstract

Sliding friction is ubiquitous in nature as are harmonic oscillators. However, when treating harmonic oscillators the effect of sliding friction is often neglected. Here, we propose a simple analytical model to include both viscous and sliding friction in common harmonic oscillator equations, allowing to separate these different types of dissipation. To compare this model with experimental data, a nanometric vibration was imposed on a quartz tuning fork, while an atomic force microscope tip was used to disturb its motion. We analyzed tuning fork resonance and ‘ring down’ experimental curves and for each case calculated the amount of sliding friction and of viscous damping, finding an agreement between the two different experiments and the model proposed.

## Introduction

Friction is an ubiquitous force that manifests from macro to nanoscales and is present almost everywhere in nature, in a wide variety of applications and extremely different industries, including automotive manufacturing, winter sports gear and nanotechnology, where new devices present a very high surface-to-volume ratio and a strong influence by surface forces. The study of friction has played an important role in the scientific community for centuries, from the formulation of the Amontons-Coulomb laws more than 300 years ago^1^, until the more recent studies of the origin of these forces at atomic scales. However, despite its universal character, friction forces still represent a challenge in the quantitative analysis of many systems. For example, its effect in the motion of an harmonic oscillators is not trivial. Despite several different approaches, from numerical ones^2^ to heuristic arguments^3^, an analytical simple solution to this problem is still lacking as demonstrated by the amount of propositions to solve it in current research^4–7^. Perhaps more notoriously this complication arises in the field of tribology.

Since the 1970s tribology has benefited from the invention of a number of new tools, such as the atomic force microscope (AFM)^8, 9^, or the quartz crystal microbalance^10, 11^, that have allowed for an unprecedented advance in the mechanistic explanation of the different friction regimes^12–18^. However, friction itself is still not completely understood, and a number of authors have used harmonic oscillators in an effort to generalize nanotribology models^11, 19–24^. Despite promising results, these approaches often lack a model allowing the analysis of a system in which there is sliding friction with a magnitude independent of the sliding speed. Some progress has been done addressing non linear friction forces, but the applicability of the models is often limited to a particular case. Commonly a simple viscous law is assumed, even if there is no particular justification for neglecting sliding friction. In this article we present simple analytical solutions to the common harmonic oscillator equations when a sliding friction force term is added. To demonstrate the validity of these solutions, an AFM tip was used to disturb the movement of a quartz Tuning Fork (TF) and its behaviour was compared to our predictions.

## Model

We focus on the effect of friction in oscillators that can still swing back and forth a few times if released from rest. Roughly speaking, if *F*_*f*_ is the friction force, *A*_*i*_ the initial displacement and *k*, *m*, *γ* the oscillator spring constant, mass and damping coefficient respectively, this work focuses on the case in which 4*F*_*f*_ < *πkA*_*i*_ and the quality factor *Q* > 2 with $Q\equiv \sqrt{km}/\gamma =k/\gamma {\omega }_{0}$.

We begin by considering steady state motion and we assume the oscillator is moving with periodic, however not simple harmonic motion due to the presence of sliding friction. The system inverts its velocity at multiples of its period *T*. As a consequence, friction is a square wave with period *T*, for which the phase can arbitrarily be chosen as *ϕ*_*friction*_ = 0. If the magnitude of the friction force is *F*_*f*_ then its Fourier series can be written as:

1 $${F}_{f}(\omega )=\frac{4{F}_{f}}{n\pi }\,\sin (n\omega t),\quad {\rm{with}}\,{\rm{n}}=1,3,5\ldots $$

where the summation over *n* is implied. Therefore, in the case where the oscillator period equals the excitation force period, the equation of motion will be:

2 $$m\ddot{x}=-k(x-{x}_{0}\,\cos (\omega t-{\varphi }_{0}))-\gamma \dot{x}-\frac{4{F}_{f}}{n\pi }\,\sin (n\omega t)\quad {\rm{with}}\,{\rm{n}}=1,3,5\ldots $$

where *x*_0_ is the excitation amplitude and *ϕ*_0_ the excitation phase, yet to be determined. The steady state solution for the equation above is:

3 $$x(t)=R\,\cos (\omega t-{\varphi }_{1})+{R}_{n}\,\cos (n\omega t-{\varphi }_{n})\quad {\rm{with}}\,{\rm{n}}=3,5,7,\ldots $$

Replacing this solution in equation (2) and solving for the Fourier components *R*_*n*_ and *ϕ*_*n*_, we find:

4 $${R}_{n}=\frac{4{F}_{f}/\pi n}{\sqrt{{(k-m{(nw)}^{2})}^{2}+{\gamma }^{2}{(n\omega )}^{2}}}$$

5 $${\varphi }_{n}=\arctan (\frac{k-m{n}^{2}{\omega }^{2}}{n\gamma \omega })$$

To find *ϕ*_1_ in equation 3 we notice that the final solution must be such that the oscillator changes the signal of the speed in phase with the friction force. One can differentiate equation (3) to find the speed, and impose zero speed each time the friction force is zero, i.e. when *ωt* = *mπ*, with m integer, leading to:

6 $$R\,\sin ({\varphi }_{1})=-n{R}_{n}\,\sin ({\varphi }_{n})$$

where again summation over n is implied. Noting that $R\,\cos (\omega t-{\varphi }_{1})$ can be written as $A\,\cos (\omega t)+B\,\sin (\omega t)$, with $A=R\,\cos ({\varphi }_{1})$ and $B=R\,\sin ({\varphi }_{1})$ we can write:

7 $$B=\sum _{n=3,5}^{\infty }\frac{4{F}_{f}}{\pi }\frac{m{n}^{2}{\omega }^{2}-k}{{\gamma }^{2}{n}^{2}{\omega }^{2}+{(k-m{n}^{2}{\omega }^{2})}^{2}}$$

To find the amplitude of the leading term $R=\sqrt{{A}^{2}+{B}^{2}}$ one only needs to find A, given by:

8 $$A=\frac{-4{F}_{f}\gamma \omega +\sqrt{{\pi }^{2}{k}^{2}{x}_{0}^{2}{Z}^{2}-{(4{F}_{f}(k-m{\omega }^{2})-\pi B{Z}^{2})}^{2}}}{\pi {Z}^{2}}$$

with

9 $$Z=\sqrt{{(k-m{\omega }^{2})}^{2}+{\gamma }^{2}{\omega }^{2}}$$

We propose below a simplified formula for the amplitude of the oscillator. It is interesting to note that for excitation frequencies close to the resonance frequency, and for oscillators that still present resonant behavior, the oscillator filters out the higher components (n = 3, 5, …) of the Fourier series. The excitation frequency appears multiplied by n in the denominator of equation (4), hence, the contribution of the higher terms becomes negligible. Consequently, the oscillator amplitude of vibration is effectively described by equation (8) with the additional simplification that *B* ≈ 0). Thus, the oscillator behaves indistinguishable from the situation where the friction force is sinusoidal.

**Figure 1:**
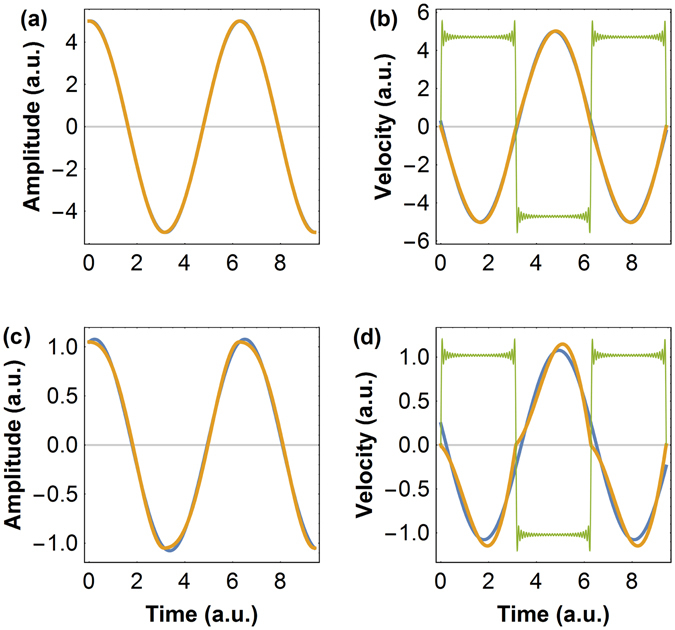
Oscillator amplitude (**a**) and velocity (**b**) versus time for a system submitted to a friction such that the oscillator gain is *G* = 5, showing the leading (blue) and the first 30 terms of the Fourier series (orange); in (**a**) the two curves are indistinguishable, in (**b**) we additionally plot the friction force (green), rescaled for clarity; (**c**,**d**) the same as in (**a**,**b**) for a higher friction force such that *G* = 1.

When excited at the resonance frequency the oscillator has a gain *G* = *R*_0_/*x*_0_, which in absence of sliding friction is simply the quality factor *Q*. Figure (1) presents amplitude and velocity calculated with only the first and with 30 terms of the Fourier series, in two limiting conditions: starting with an oscillator with a gain *G* = 10^4^ (=*Q*), friction is increased such that the oscillator gain becomes *G* = 5 (Fig. (1)(a,b)) and *G* = 1 (Fig. (1)(c,d)). These cases correspond to friction forces such that 4*F*_*f`*_ ≈ *πkx*_0_. Figure (1) illustrates that even for overdamping friction forces and very small oscillator gains *G*, equation (8), which neglects the higher order terms of the Fourier series, is very effective at describing the system. For very low vibration amplitudes, smaller than the excitation amplitude *x*_0_, the first term alone starts to be insufficient to describe the system. Additionally, for such extreme cases one would certainly need to consider also static friction i.e when the oscillator speed is zero, as it may rest at zero speed for a time longer than in the harmonic situation. In fact, we suggest that the effect of static friction in harmonic oscillators can also be treated using an appropriate Fourier series.

Equation (8) is generally difficult to fit to experimental data. However this equation looks like and can be approximated to a Lorentzian curve, which may be deceiving, because one can erroneously conclude from a Lorentzian shaped curve that all force involved are linear. Nonetheless it is useful to express it in terms of a Lorentzian dependence which is routinely fitted to resonance curves.

From equation (8) it follows that at resonance the amplitude *R*_0_ is given by:

10 $${R}_{0}=Q({x}_{0}-\frac{4{F}_{f}}{\pi k})={x}_{f}{Q}_{f}$$

where *x*_*f*_ and *Q*_*f*_ are defined as the effective parameters obtained from a Lorentzian fit, *Q* being the quality factor defined such that it depends on damping but not on sliding friction. Similarly, for a given amplitude *R* one can define an effective damping *γ*_*f*_, by adding the leading term of the sliding friction series to the term containing the damping coefficient γ in equation (2):

11 $${\gamma }_{f}=\gamma +\frac{4{F}_{f}}{\pi {\omega }_{0}R}$$

The amplitude *R* must be proportional to *x*_0_ and to the effective quality factor *Q*_*f*_ ≡ *k*/(*γ*_*f*_ *ω*_0_); thus putting *R* = *ax*_0_ *k*/(*γ*_*f*_ *ω*_0_), with a a proportionality constant, and solving for equations 10 and 11 one finds:

12 $${\gamma }_{f}=\gamma {\mathrm{(1}-4{F}_{f}/a\pi k{x}_{0})}^{-1}$$

or equivalently,

13 $${Q}_{f}=Q\mathrm{(1}-4{F}_{f}/a\pi k{x}_{0})$$

and

14 $${x}_{f}={x}_{0}\frac{1-4{F}_{f}/\pi k{x}_{0}}{1-4{F}_{f}/ak\pi {x}_{0}}$$

Consequently, the solution presented in equation (8) can be written in the usual form:

15 $$R=\frac{{x}_{f}{\omega }_{0}^{2}}{\sqrt{{({\omega }_{0}^{2}-{\omega }^{2})}^{2}+{(\frac{\omega {\omega }_{0}}{{Q}_{f}})}^{2}}}$$

Finally, comparing equations 8 and 15 yields *a* ≈ 4/3, allowing to calculate *x*_*f*_ and *Q*_*f*_.

We turn now to a common situation where the oscillator is left to stop after released from an initial amplitude, *A*_*i*_. From the previous analysis one concludes that the resonance frequency is not affected by pure sliding friction forces. Additionally we know that an harmonic oscillator vibrates at its resonance if released from rest. Consequently, as before, we consider the friction force as a square wave with frequency equal to the resonance frequency *ω*_*r*_. The situation can be described by the following equation:

16 $$m\ddot{x}+\gamma \dot{x}+kx=\frac{4{F}_{f}}{\pi }\,\sin ({\omega }_{r}t)$$

where the higher order terms have been neglected for reasons explained earlier. Equation (16) must be solved with initial conditions *x*(0) = *A*_*i*_. Obviously, this equation only describes the system until its amplitude is zero, as for instants after that the equation above makes no sense. The solution resulting from considering $4{Q}^{2}\gg 1$, is:

17 $$x(t)=[\frac{-4{F}_{f}}{\pi \gamma {\omega }_{r}}+({A}_{i}+\frac{4{F}_{f}}{\pi \gamma {\omega }_{r}}){e}^{-\gamma t/2m}]\cos ({\omega }_{r}t)$$

Unlike the common harmonic oscillator, the amplitude decay is not just a simple exponential, and an ‘offset’ appears as a signature of sliding friction. This equation provides a very simple way to test if the system is subject to sliding friction since in that case, Log[*v*(*t*)] does not give a straight line.

The same expression can be deduced in a much simpler way. Considering the system with an initial kinetic energy $1/2\,m{v}_{i}^{2}$ and at the equilibrium position, after a certain time the oscillator has gone forth and returned to the initial position, losing an energy 2*F*_*f*_ *A* due to sliding friction and an energy (*π*/2)*γAv* due to damping. We can approximate *v* such that $v(t)=v\,\sin ({\omega }_{r}t)$ between instant *i* and *f*. Thus, during the time it goes back and forth, the oscillator loses energy according to:

18 $$\frac{1}{2}m({v}_{i}^{2}-{v}_{f}^{2})=2{F}_{f}\frac{v}{{\omega }_{r}}+\frac{\pi }{2}\gamma {v}^{2}$$

Since we are considering a small time interval *dt* = *π*/*ω*_*r*_, one can approximate *v*_*i*_ = *v* + *dv*/2 and *v*_*f*_ = *v* − *dv*/2. To calculate the rate at which the system is losing energy one must divide equation (18) by the time *dt* it takes the system to go back and forth. Equation (18) becomes:

19 $$-m\frac{dv}{dt}=\frac{2{F}_{f}}{\pi }+\frac{\gamma v}{2}$$

The solution to this differential equation when solved with initial conditions *v* = (*A*_*i*_ *ω*_*r*_) is identical to the envelope of equation (17). The method presented here can also be used to calculate the effect of friction in oscillators for which the damping is proportional to the square of the speed. For that one needs only to recalculate the energy lost due to damping and replace it in equation (18).

**Figure 2:**
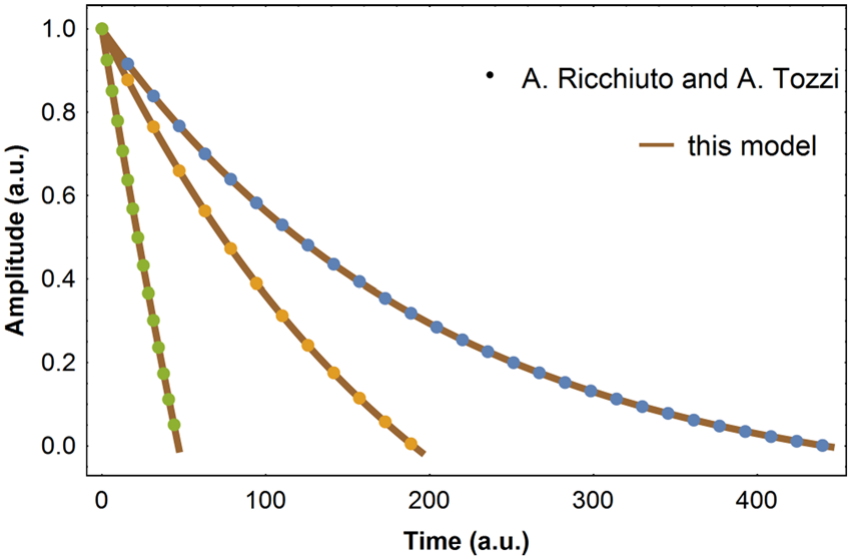
Oscillator amplitude versus time. The oscillator is submitted to three different sliding friction forces. The amplitude decay is exponential with a negative asymptote.

A different solution has been obtained by A. Ricchiuto and A Tozzi^25^ by solving iteratively as many differential equations as half cycles and then imposing continuity. Here, instead we compute the energy loss and continuously distribute this energy over time. For comparison we show in Fig. (2) a plot of both solutions for the same oscillator with different sliding friction forces. Even though the formulas are different they quantitatively reproduce the same trends.

## Experimental Results

To experimentally verify the validity of this model, an experimental setup predominant in the study of friction with oscillators^19–21, 23^, detailed in the Methods section, was used.

**Figure 3:**
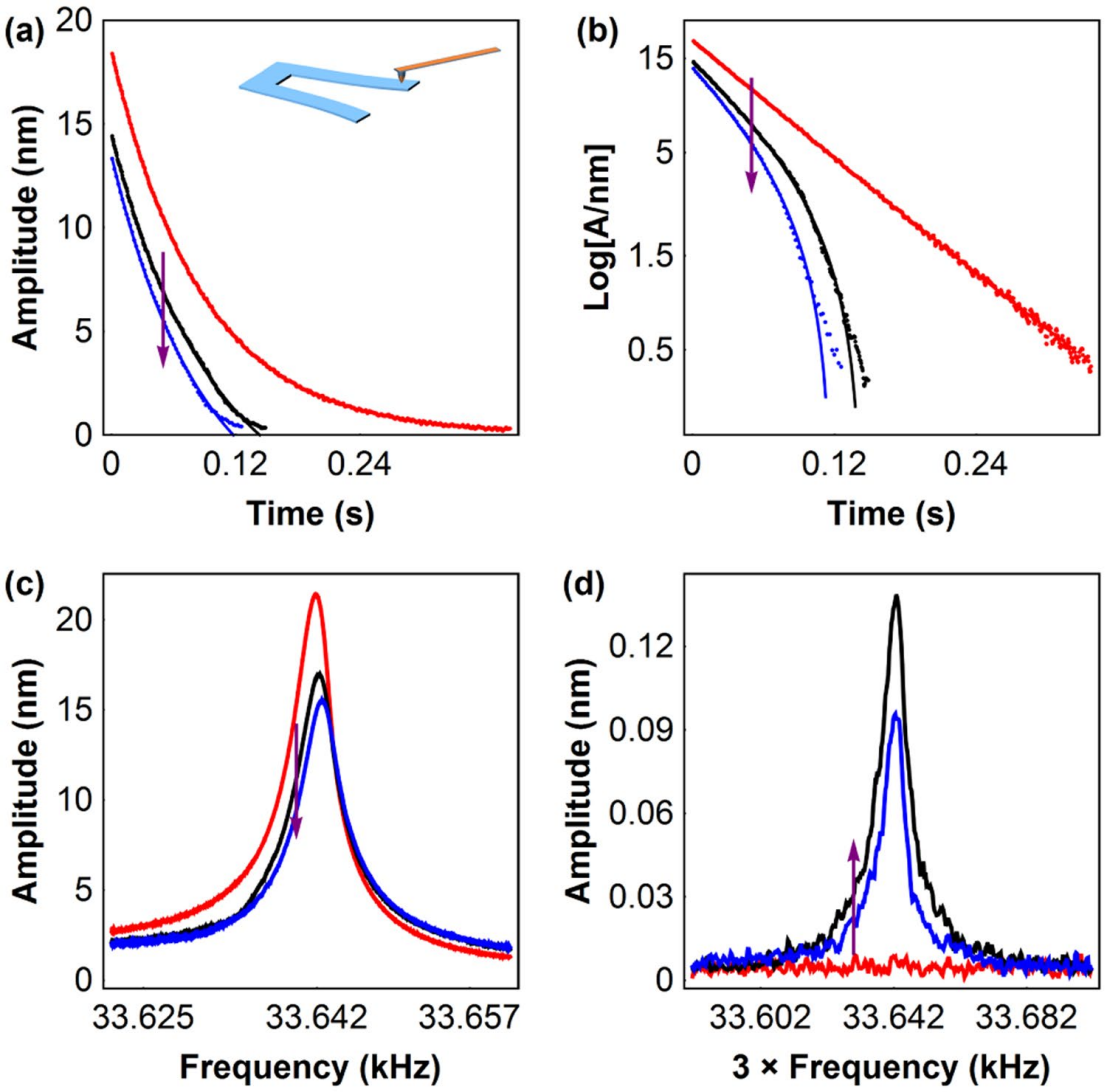
Driving a quartz tuning fork while applying a normal load (0–10 nN): (**a**) and (**b**) ring down experiments, where the excitation is turned off and the oscillator speed decays. Experimental data is presented as solid lines and fits of equation (17) are presented as dots. (**c**) Frequency response of the oscillator with different applied loads. (**d**) Frequency response of the oscillator measured around ω_0_, when driving it near *ω*_0_/3. All experiments were performed using similar loads. The red curve corresponds to the free oscillator and the arrow represents increasing load.

The most straightforward way to test the assumption of a viscous friction force law is to perform ‘ring down’ experiments as described previously. A controlled normal load (on the order of a few nN) was applied by the AFM tip to a mechanically excited quartz tuning fork (TF). As this excitation was turned off, the amplitude of oscillation was monitored until the oscillator stopped. Figure (3)(a,b), present the results of this experiment for the free oscillator (red curve) and for two different applied loads (black and blue). In the unperturbed oscillator, the decay is exponential, evidenced by the linear evolution of Log[$\dot{x}$] with time. This indicates a viscous damping of the TF, as expected. However, for non-zero applied loads as small as 5 nN, the oscillator yields a different ring down decay, as seen by the black and blue curves in these figures. Experimental data was fitted using equation (17), and the resulting fits are also plotted on the figures. An excellent agreement between experiment and this model was found down to very small oscillating speeds, where the tip may be sticking to the TF and the model is not valid anymore, evidenced by the last few points of Fig. (3)(a,b). Additionally we measured the frequency response of the oscillator at different applied loads. Figure (3)(c) presents the results of this experiment, where the excitation frequency was scanned around *ω*_0_ and the amplitude of oscillation recorded, while controlling the normal load. As evidenced in this figure, all curves can be fitted quite well by a Lorentzian dependence. From the fits of these curves one can extract *x*_*f*_ and *γ*_*f*_ and compute *F*_*f*_ and *γ* from equations 12 and 14. The results of these fittings from different ring down and resonance curve experiments, at similar loads, can be found in Table 1.

**Table 1:** Fitting parameters extracted from several ring down and transfer function experiments, with different normal loads applied to the oscillator.

~Load (nN)	*F*_*f,rd*_ (nN)	*F*_*f,res*_ (nN)	*γ*_*rd*_ (*μ*Nm^−1^s)	*γ*_*res*_ (*μ*Nm^−1^s)
Free	0	0	22	20
9	15	16	20	18
13	17	16	23	20

Suffix ‘rd’ represents ‘ from ring down curves’ and ‘res’ represents from ‘resonance curves’.

## Discussion

The fitting parameters from either ring down experiments and resonance curve measurements demonstrate that the two experiments are compatible and agree well in describing the friction force the oscillator is experiencing. Noting that speed is changing in both experiments, the fact that the models fit the experimental data means that the role of velocity within this range of velocities is well taken into consideration.

Furthermore, and perhaps more importantly, these independent measurements demonstrate that there is no significant change in the damping coefficient *γ*, indicating sliding friction as the main mechanism of energy loss. In fact, we have performed a number of experiments using both methods, with different TFs, cantilevers and loads, and no significant increase of the damping coefficient due to the interaction was detected. For systems akin to ours, the effect of a constant friction force cannot be ignored, even in cases where the oscillator transfer function can be fitted as a Lorentzian. Accurate quantitative results can only be obtained if one includes sliding friction.

Looking at equation (4), it becomes quite tempting to conclude that the oscillator can be very effectively excited by driving it at odd subharmonics of the resonance frequency. The oscillator will work out to filter the terms that do not excite it at its resonance frequency. Experimentally, this works remarkably well particularly for an excitation frequency of *ω*_0_/3. Figure (3) shows the response of the oscillator around the resonance frequency *ω*_0_ but excited at *ω*_0_/3 in the absence of friction and for two different loads. Note that in this case the equations developed here do not apply because our initial hypothesis that the friction frequency was equal to the excitation frequency is not valid anymore. Even though we were able to excite the TF at frequencies down to *ω*_0_/9 measuring a resonant response at *ω*_0_, the fact is that if the amplitude is significant at n times the excitation frequency, then friction is necessarily not a square wave with frequency equal to the excitation frequency. Actually it is no longer a square wave. Nonetheless, the fact that the oscillator can be excited at *ω* = *ω*_0_/3 provides remarkable evidence that the oscillator experiences friction as a series of harmonic terms.

In conclusion, we have provided a quantitative method to simultaneously extract the damping and the often forgotten sliding friction on experiments involving harmonic oscillators. We have developed two independent analytical methods to analyze the effect of these dissipation mechanisms on the movement of an oscillator such as a TF, and compared our predictions with experiment using an AFM tip and a TF, in a common experimental configuration often used in nanotribology. This model in conjunction with experimental results demonstrate that it is not realistic to assume a simple viscous friction law based solely on the fact that the oscillator response is of the Lorentzian type.

Ring down decay experiments exhibit clearly the presence of sliding friction even when in the same conditions the resonance curve is rather Lorentzian like.

We stress, however, that this model is not limited to nanoscale friction, but can be applied to even the most simple experiments performed in a physics class, providing a simple and analytical answer to a common problem pervasive in nature, where friction and oscillatory movement are often present but overlooked.

## Methods

The experimental setup consists of a nanosized AFM tip that exerts a force on an harmonic oscillator, in this case a common wrist watch quartz tunning fork, with nominal resonant frequency of 2^15^ Hz. The symmetric vibration mode of the TF is mechanically excited with a piezoelectric dither and the TF oscillation is measured while the AFM tip applies a constant force on one of its prongs. The system is then defined as a silicon tip sliding on a bare quartz surface. A sketch of the setup can be seen on the inset of Fig. (3)(a). The TF was excited such that its free amplitude of oscillation at *ω*_0_ was close to 25 nm.

The movement of the TF was detected using a *Stanford Research Systems* SR850 DSP Lock-in Amplifier. To control the force exerted on the quartz surface we used a custom-made AFM system, the Force Feedback Microscope^26, 27^, that employs an interferometric detection system^28^ and a counteracting force feedback loop to keep the AFM tip position constant and measure the tip-surface interaction^29, 30^. To measure the deflection of the cantilever we used a Thorlabs TLS001-635 laser source, a Thorlabs DET100A/M photodetector and a *FEMTO* DLPCA-200 transimpedance amplifier.

The AFM cantilever used for these experiments was the Bruker Microlever MLCT-F, with nominal spring constant of 0.6 N/m and nominal tip radius of 20 nm.

### Data Availability

The datasets generated during and/or analyzed during the current study are available from the corresponding author on reasonable request.
